# Biogas production experimental research using algae

**DOI:** 10.1186/s40201-015-0169-z

**Published:** 2015-03-13

**Authors:** Pranas Baltrėnas, Antonas Misevičius

**Affiliations:** Institute of Environmental Protection, Vilnius Gediminas Technical University, Vilnius, Lithuania

**Keywords:** Biogas, Methane, Algae, Batch reactor, Anaerobic digestion

## Abstract

The current study is on the the use of macro-algae as feedstock for biogas production. Three types of macro-algae, *Cladophora glomerata* (CG), *Chara fragilis* (CF), and *Spirogyra neglecta* (SN), were chosen for this research. The experimental studies on biogas production were carried out with these algae in a batch bioreactor. In the bioreactor was maintained 35 ± 1°C temperature. The results showed that the most appropriate macro-algae for biogas production are *Spirogyra neglecta* (SN) and *Cladophora glomerata* (CG). The average amount of biogas obtained from the processing of SN – 0.23 m^3^/m^3^d, CG – 0.20 m^3^/m^3^d, and CF – 0.12 m^3^/m^3^d. Considering the concentration of methane obtained during the processing of SN and CG, which after eight days and until the end of the experiment exceeded 60%, it can be claimed that biogas produced using these algae is valuable. When processing CF, the concentration of methane reached the level of 50% only by the final day of the experiment, which indicates that this alga is less suitable for biogas production.

## Introduction

Although algae are beneficial to the production of oxygen, but their rapid spread to minus. The spread of algae in water bodies is an important environmental problem, which results in disappearance of the water bodies. After the review of the Lithuanian and foreign scientific literature on the use of algae, it can be noted that algae are mainly used for the production of biofuels including biogas, in medicine and in food industries [1-3].

Algae can be unicellular, multicellular, and acellular. They can live alone or in colonies. Algae can be divided into micro-algae (microscopic cells 3-10μm in size) and macro-algae. Depending on the type of algae, usually a half of its nutritional value is protein, less than a third - carbohydrates, and the remaining compounds are fats, vitamins and trace elements. Factors influencing more like algal production are:

- High water temperature (depends on solar radiation, topography, currents, water sources);
- Nutrients (depend on the ecosystem, sediments, and the shoreline);
- Water pH;
- Low oxygen levels;
- Anthropogenic pollution;
- The physical parameters of the water body (the speed of current, the cross-section area, the discharge, the steepness of coasts);
- Soil for entrenchment;
- Other factors (including the mechanic effect of sediments, bottom shifts, species and the spread of plants, fauna).

The anaerobic processing of organic waste and its utilization for energy production is encouraged by stringent environmental regulations, growing waste disposal costs and rising prices of energy resources [4]. The use of algae as a feedstock could address two major environmental problems. First, to use algae as a feedstock for biogas production, it should be extracted from water, thus, the water body would be cleaned and, at the same time, protected from eutrophication. Second, the air pollution would be reduced and the climate change stabilized. The biological decomposition of organic waste produces gases that consist of methane, carbon dioxide, hydrogen, hydrogen sulphide. Methane is the gas that has the 21-times-stronger impact on the greenhouse effect than carbon dioxide [5-7]. Having these two issues solved could, at the same time, produce economic benefits. Biogas, obtained during the bioreactor treatment of accumulated algae, could be used as fuel for the production of electricity and/or thermal energy [8,9].

Razon and Tan [1] cultivated algae in the photo-bioreactors and used one part of the cultivated algae for biodiesel production and the other part for biogas production [1]. Harun et al. [10] analyzed the possibility of using micro-algae for biogas, biodiesel and bioethanol production. These researchers argue that it is most useful to process algae for biogas and biodiesel production, because most of the energy, i.e. 16.14 MJkg^-1^, is produced when doing so [10]. Zhong et al. [11] studied the biogas productivity with five organic waste mixtures, i.e. blue algae and corn silage. The scientist argues that the best yield of biogas is when the C/N (carbon/nitrogen) ratio is 20/1; then the biogas production rate is 0.6 m^3^/kg DW, and methane concentrations were about 55% [11]. Mussgnung et al. [12] conducted biogas production experimental studies with six species of micro-algae [12]. The results of the studies demonstrate that the biogas production rate is from 0.28 to 0.65 m^3^/kg DW (DW – dry weight) and methane concentrations range from 54 to 67% [12]. Salermo et al. [13] have also conducted biogas production studies using algae, however, the scientists have been mixing algae in different proportions with soybean oil and glycerin [13]. The amount of biogas ranged from 0.3 to 0.5 ml/mld, and methane averaged in 68% [13]. Vergara-Fernandez et al. [14] used two types of algae for biogas production by dissolving the algae in the batch bioreactor. Their results indicate that biogas production reached 0.18 m^3^/kg DW, and methane concentration was 65% [14]. The analysis of literature shows that various types of micro-algae are used for biogas production. However, there is no information on the use of freshwater macro-algae for biogas production. It is therefore relevant to examine the efficiency of macro-algae for energy production.

The aim of the current research is to analyze what amount of biogas and its main component -methane - is emitted during the anaerobic processing of three types of macro-algae at the mesophilic mode in the batch bioreactor. Also, the research aims at identifying which type of the three tested algae species is the most efficient raw material for biogas production.

## Materials and methods

### Waste characteristics

The amount of used algae for the analysis and algae moisture are presented in Table 1. The same amount of each type of algae, i.e. 2000 grams, was taken for the analysis. The dry material volume in the bioreactor was calculated measuring algae moisture according to the formula.

**Table 1:** **Algae, quantity and moisture content**

**Algae**	**Moisture content, %**	**Dry matter, g/kg**	**Amount of algae in bioreactor, g DW**
*Cladophora glomerata*	92.4	76	152
*Chara fragilis*	90.6	94	188
*Spirogyra neglecta*	90.1	99	198

DW – dry weight.

### Experimental set-up

**Figure 1:**
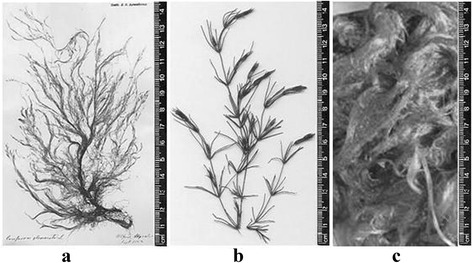
**Macro algae (a** ***– Cladophora glomerata*** **, b –** ***Chara fragilis*** **, c** ***– Spirogyra neglecta*** **).**

Figure 1 present the algae which were carried out experimental studies of biogas production. *Cladophora glomerata* [15], *Chara fragilis* [16] *Spirogyra neglecta* algae were selected according to their occurrence in water bodies. Distribution of algae in water bodies is one of the biggest.

**Figure 2:**
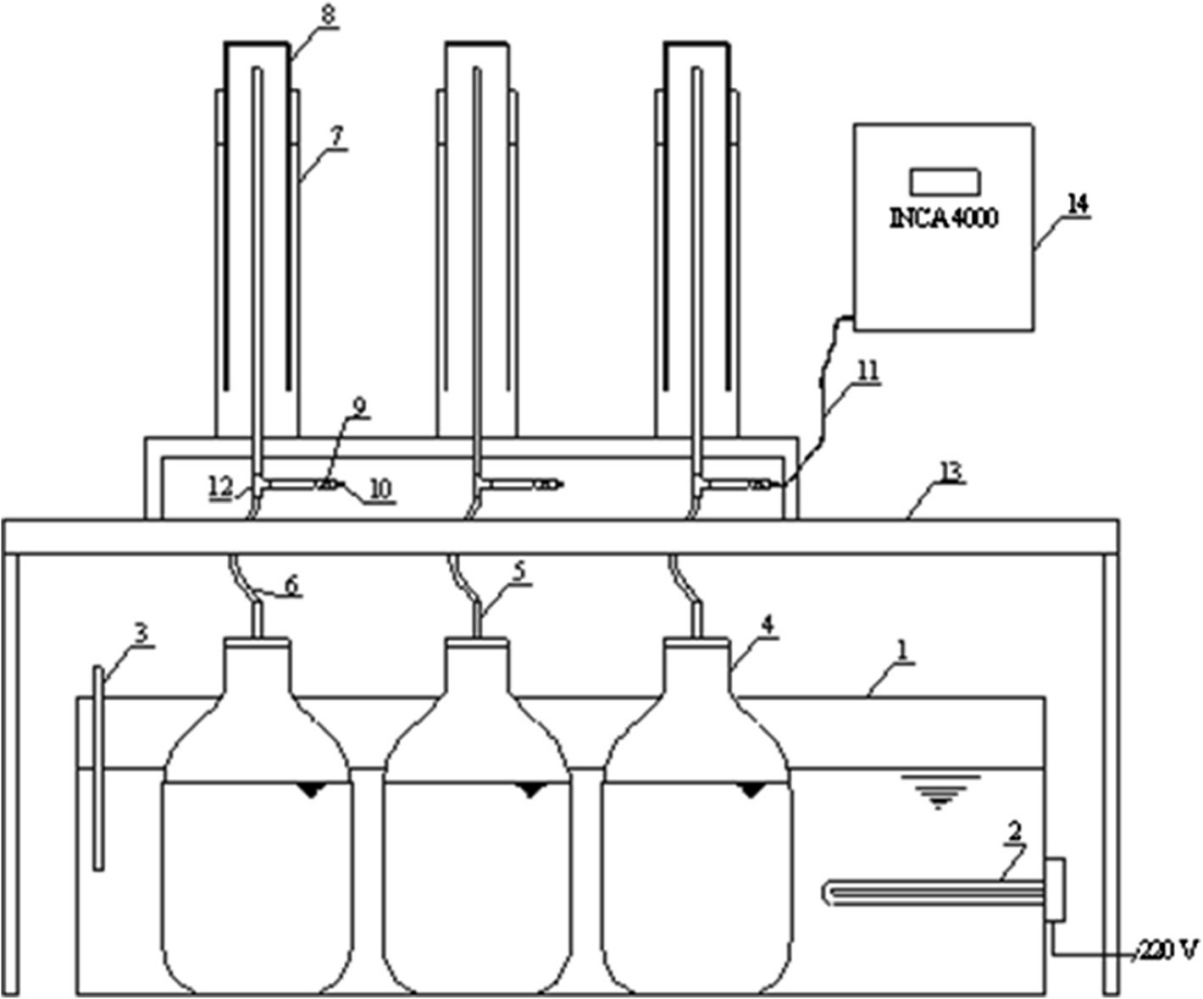
**Scheme of the batch bio-reactor stand: 1 – vessel with water, 2 – substrate heating device, 3 – temperature sensor, 4 – bio-reactor, 5 – branch pipe for hose fixing, 6 – flexible hose, 7 – vessel with water, 8 – biogas accumulation vessel (PVC pipe), 9 – valve, 10 – branch pipe for gas discharge, 11 – elastic hose, 12 – T-socket, 13 – table, 14 – gas analyser.**

The analyses were carried out in batch reactors (Figure 2) with a total volume of 5 L and a the working volume of 3 L (in the bioreactor substrate amounted to 3 L). The bioreactors, which worked at the mesophilic mode, were filled with three species of algae and were maintained at 35 ± 1°C. Mesophilic mode was chosen because it is the most optimal and most frequently not only in the laboratory but also in industrial bioreactors. At this temperature mode of substrates digestion is more stable and faster [17].

### Reactor operation

The batch bioreactors were loaded with substrate through substrate intake; the substrate was removed through the same opening after the experiment (Figure 2).

In the bioreactor was maintained at 35 ± 1°C temperature. To know the current temperature in the container with water, the temperature gauge was built-in. If the temperature falls below the set temperature, the temperature sensor goes off and the electronic heating element automatically heats the water in the container and the substrate in the bioreactor to the required temperature. This type of bioreactor does not have any mixing equipment; also, because of the low volume mixing is not necessary. Bioreactor every day was mechanically shaken. The bioreactor is placed on the shaker and shake for 10-15 minutes, the substrate is evenly mixed. To know the amount of gas, 4,5 liter biogas accumulation vessel, hoses and sleeves for fixing, and a ruler, was constructed. The biogas storage tank was connected to the biogas analyzer by means of the flexible hose. When the device is turned on, the biogas storage tank valve is opened simultaneously to allow biogas flow into the analyzer. The biogas composition measurement lasts nine minutes. At the end of measuring, the device presents the average biogas composition values.

### Analytical methods

For the measurement of moisture, 10 grams of the test material was weighed. The material was placed in the thermostatic drying box and was dried at 105°C ± 2°C for three hours. After drying, the container with the test material was placed in the dessicator for 45 minutes to cool down. After the test material cooled down, its moisture was calculated according to the formula:

1 $$ Wa=\left(\left(Gw- Ga\right)/ Ga\right)\cdotp 100\% $$

here:

- *Wa* – material moisture content,%;
- *Gw* – wet weight of the material, g;
- *Ga* – absolute dry weight, g.

Measurements were carried out with the analyser INCA 4000 which measures methane (%), carbon dioxide (%) oxygen (%) concentration and the concentration of hydrogen sulphide in (ppm). The measurable ranges are: for oxygen – 0–25% (±1%); hydrogen sulphide – 0–100 ppm (±5%); methane – 0–100% (±1%); carbon dioxide – 0–100% (±1%). The analyser INCA 4000 works at an ambient air temperature range from -5°C to +40°C, and relative humidity from 0 to 95%. Substrate pH was determined at the beginning and end of the experiment using. pH meter measuring range from 0 to 14 (±0.1) units.

## Results and discussion

**Figure 3:**
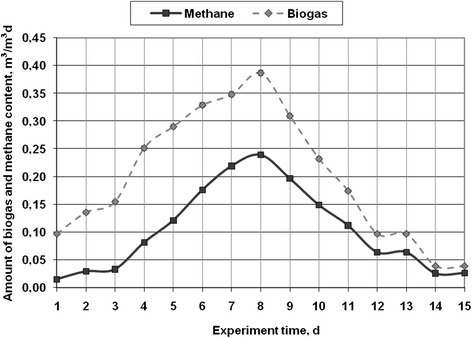
**The amount of biogas (m** ^**3**^ **/m** ^**3**^ **d), methane content (m** ^**3**^ **/m** ^**3**^ **d) in the anaerobic digestion of macro algae -** ***Cladophora glomerata*** **under batch reactor mesophilic conditions.**

**Figure 4:**
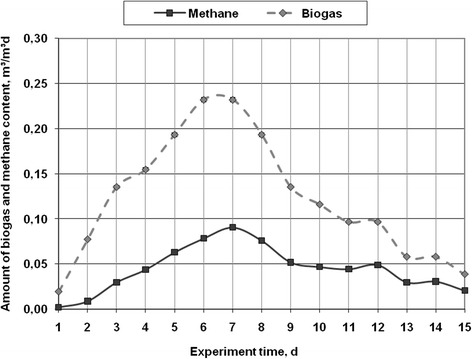
**The amount of biogas (m** ^**3**^ **/m** ^**3**^ **d), methane content (m** ^**3**^ **/m** ^**3**^ **d) in the anaerobic digestion of macro algae -** ***Chara fragilis*** **under batch reactor mesophilic conditions.**

**Figure 5:**
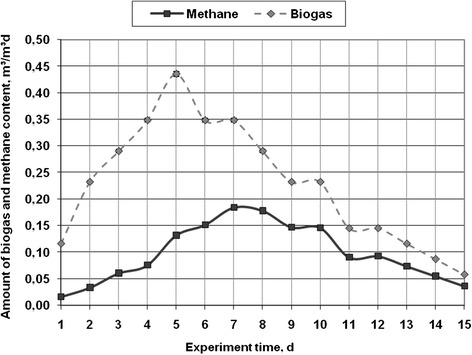
**The amount of biogas (m** ^**3**^ **/m** ^**3**^ **d), methane content (m** ^**3**^ **/m** ^**3**^ **d) in the anaerobic digestion of macro algae -** ***Spirogyra neglecta*** **under batch reactor mesophilic conditions.**

Three experimental 15-days studies with macro-algae were conducted at 35 ± 1°C. The results of the studies are presented in Figures 3, 4, and 5. The amount of biogas and its main component – methane – are presented in the results. Although other components were measured, their graphic results are not presented, but are mentioned in the text. As shown in Figure 3, the biogas production had a tendency to rise up to day 8 of the experiment and reached 0.387 m^3^/m^3^d. Later, the amount of biogas decreased to 0.039 m^3^/m^3^d at the end of the experiment. The experimental studies was conducted using 152 g of dry weight of macro-algae - *Cladophora glomerata* - can produce 0.06 m3 of biogas during 15 days. Each plant or waste type has its own organic composition. In terms of the anaerobic processing, the biomass is evaluated with respect to the amount of fat, protein and carbohydrates in it. Given different quantities of these elements, a different amount of biogas and the concentration of methane is obtained. If there are more carbohydrates in the substrate, the biogas production process is faster, because of the faster decomposition of carbohydrates, and the concentration of methane can reach 50-60% [18]. However, if there is more fat or protein, the biogas production is slower, but the concentration of methane is higher. It can range from 75-90% [19]. Algae are the source of oils, carbohydrates and protein. Therefore, by knowing the chemical composition, the biogas and methane production capacity can be determined. In this case, with regard to the concentration of methane in biogas, it can be assumed that during the processing of *Cladophora glomerata*, the amount of protein was the biggest, because the concentration of methane was 70%. The decrease of biogas may occur due to several reasons. Without the supply of substrate in bioreactor, microorganisms do not get the nutrients, as a result, the biogas production is slowing down. Therefore, it can be concluded that on the 8-9 day of the experiment it is necessary to add some substrate. pH could also change, as at the beginning of the experiment the pH value was 6.8 ± 0.1, but at the end it decreased to 6.3 ± 0.1. When pH drops below 6.5, the biogas and methane production slows down [20-22]. In biogas the amount of oxygen ranged from 4.2% at the beginning of the experiment, and then fell, reaching only 0.1%. The concentration of hydrogen sulfide in biogas varied in the range from 3 ppm to 35 ppm, therefore, the biogas purification of this pollutant is not necessary.

The anaerobic decomposition of *Chara fragilis* in the bioreactor, biogas and methane variation was similar to the previously mentioned case. The emission of biogas increased from 0.019 m^3^/m^3^d at the beginning of the experiment to 0.232 m^3^/m^3^d on day 6. Then, the amount of biogas was gradually decreasing until the final day of the experiment. Until day 7 of the experiment, the concentration of methane was increasing and reached 0.09 m^3^/m^3^d (i.e. 38.9% of the biogas volume), later, the concentration was stable for several days, and since day 10, was increasing again and reached 0.021 m^3^/m^3^d (53.1% of the biogas volume) on the final day of the experiment. Compared to other research results [1,2,10], the results of the current research suggest that during the processing of macro- and micro-algae, the concentration of methane in biogas in some cases is similar, and in some cases the obtained concentration is 10-15% higher. This fluctuation may occur due to the protein and carbohydrate amount in algae. Given the fact that the experiment lasted 15 days, it can be maintained that algae decomposed much faster during the anaerobic processing. The faster decomposition is conditioned by the fact that the walls of algae are easily destroyed, there is almost no lignin, thus algae is more technological in the production of biogas as compared with terrestrial plants [23]. The level of pH at the beginning of the experiment was 6.5 ± 0.1, while at the end it dropped to 6.2 ± 0.1. The anaerobic decomposition process was also influenced by pH. In biogas the amount of oxygen ranged from 2.8% at the beginning of the experiment, and later, at the end, it dropped to 0%. The concentration of hydrogen sulfide in biogas varied in the range from 24 ppm to 60 ppm, therefore, the biogas purification of this pollutant is not necessary.

During the processing of the macro-algae - *Spirogyra neglecta* - the upward trend in the emissions up to day 5 and then a downward trend until the end of the experiment could be observed. At the beginning of the experiment, the amount of biogas reached 0.12 m^3^/m^3^d, later, it increased to 0.44 m^3^/m^3^d and at the end of the experiment it dropped to 0.06 m^3^/m^3^d. In this case, the decrease in biogas amount can be observed after day 5 of the experiment, then, the decreased amount remains the same; later, it decreases again and the process repeats three times. This trend may occur due to the intensity change of the acetogenesis and methanogenesis processes [24]. The bacteria that produce methane during the process of methanogenesis cannot decompose acetates produced during the acetogenesis process. Therefore, when the amount of acetates increases, the substrate acidifies, and slows down the biogas production process. At the beginning of the experiment, the level of pH was 6.4 ± 0.1, while at the end it dropped to 6.2 ± 0.1.

The concentration of methane in biogas was increasing since day 4 at the rate of 10% up to day 8 of the experiment and that day it reached 0.178 m^3^/m^3^d (i.e. 61.3% of biogas volume). Since day 8, the concentration of methane stabilized and until the final day of the experiment only slight change in the concentration could be observed. The range is from 0.036 m^3^/m^3^d to 0.092 m^3^/m^3^d. Biogas, containing such levels of methane, is a valuable fuel and can be used for the production of energy or as a motor fuel, as the concentration of methane is 60% [25]. At the beginning of the experiment, the level of pH reached 6.4 ± 0.1, while at the end it dropped to 6.35 ± 0.1. During the experiment, the anaerobic conditions were secured, as the amount of oxygen ranged from 3.1% at the beginning of the experiment and later, at the end, it dropped to 0.1%. The concentration of hydrogen sulfide in biogas varied in the range from 15 ppm to 72 ppm, therefore, the biogas purification of this pollutant is not necessary.

## Conclusions

1. The biggest average amount of biogas was measured during the processing of macro-algae *spirogyra neglecta* and reached 0,23 m^3^/m^3^d.
2. The highest average methane concentration in biogas was gauged during the decomposition of *cladophora glomerata* and reached 51,4%; during the processing of other algae (*spirogyra neglecta* and *chara fragilis*), the gauged concentration of methane was 46,5% and 36,9% respectively.
3. The assessment of both parameters, i.e. the amount of biogas and methane concentration, indicates that the most suitable algae for the production of energy are both *spirogyra neglecta* and *cladophora glomerata*.
4. During all the conducted experiments of processing all three kinds of algae, the anaerobic conditions were secured, and hydrogen sulfide concentrations were below 100 ppm. Therefore, biogas purification of this pollutant is not necessary.
5. The comparison of the emitted biogas and methane concentration during the processing of macro-algae with other biodegradable waste indicates that macro-algae is a suitable raw material for the production of biogas. During the processing of algae, the amount of biogas fluctuates between 0,64 and 1,32 m^3^/m^3^d, and the volume of methane ranges from 0,24 to 0,68 m^3^/m^3^d. During the decomposition of other biodegradable waste, the amount of biogas reaches from 0,9 to 1,67 m^3^/m^3^d. However, the concentration of methane is lower as compared with that obtained from macro-algae.
